# Composition and evolutionary characterization of the gut microbiota in pigs

**DOI:** 10.1007/s10123-023-00449-8

**Published:** 2023-11-20

**Authors:** Shuhong Zhang, Huan Zhang, Cheng Zhang, Guan Wang, Chuanxing Shi, Zhiqiang Li, Fengyi Gao, Yanyan Cui, Ming Li, Guangli Yang

**Affiliations:** 1 College of Biology and Food, Shangqiu Normal University, Shangqiu, 476000 China; 2 College of Animal Science and Technology, Henan Agricultural University, Zhengzhou, 450002 China

**Keywords:** Pigs, Gut microbiota, Composition and function, Composition and evolution, Phylogeny

## Abstract

The intestinal microbiota plays significant role in the physiology and functioning of host organisms. However, there is limited knowledge of the composition and evolution of microbiota-host relationships from wild ancestors to modern domesticated species. In this study, the 16S rRNA gene V3–V4 in the intestinal contents of different pig breeds was analyzed and was compared using high-throughput sequencing. This identified 18 323 amplicon sequence variants, of which the *Firmicutes* and *Actinobacteria* phyla and *Bifidobacterium* and *Allobaculum* genera were most prevalent in wild pigs (WP). In contrast, *Proteobacteria* and *Firmicutes* predominated in Chinese Shanxi Black pigs (CSB), while *Firmicutes* were the most prevalent phylum in Large White pigs (LW) and Iberian pigs (IB), followed by *Bacteroidetes* in IB and *Proteobacteria* in LW. At the genus level, *Shigella* and *Lactobacillus* were most prevalent in CSB and LW, while *Actinobacillus* and *Sarcina* predominated in IB. Differential gene expression together with phylogenetic and functional analyses indicated significant differences in the relative abundance of microbial taxa between different pig breeds. Although many microbial taxa were common to both wild and domestic pigs, significant diversification was observed in bacterial genes that potentially influence host phenotypic traits. Overall, these findings suggested that both the composition and functions of the microbiota were closely associated with domestication and the evolutionary changes in the host. The members of the microbial communities were vertically transmitted in pigs, with evidence of co-evolution of both the hosts and their intestinal microbial communities. These results enhance our understanding and appreciation of the complex interactions between intestinal microbes and hosts and highlight the importance of applying this knowledge in agricultural and microbiological research.

**Supplementary information:**

The online version contains supplementary material available at 10.1007/s10123-023-00449-8.

## Introduction

The gut microbiota plays a major role in the overall health of mammals (Brestoff and Artis 2013). Domestic pigs (*Sus scrofa*) diverged from their wild ancestors in Eurasia approximately 10,000 years ago, and the selection of specific traits has resulted in significant phenotypic changes (Larson et al. 2005; Rubin et al. 2012). Pigs are used extensively as model animals in research on human diseases, development, and responses to infection (Lunney et al. 2021). The pig is also useful for investigating the evolution of the gut microbiota in a species as both the wild ancestor and a variety of domesticated breeds can be used for comparison (Ushida et al. 2016).

There have been a variety of recent investigations into the gut microbiota of the pig, many concerned with agricultural traits and applications (Crespo-Piazuelo et al. 2018; Gao et al. 2019; Kelly et al. 2017; Xiao et al. 2018; Yang et al. 2016; Zhao et al. 2015), such as weight gain (Mach et al. 2015; Ramayo-Caldas et al. 2016) and food intake and conversion (Camarinha-Silva et al. 2017; McCormack et al. 2017; Quan et al. 2018, 2019; Yang et al. 2017). Several studies have also reported on microbial composition in different intestinal regions. These studies have mostly been restricted to specific pig breeds, including the Large White (Zhao et al. 2015), Laiwu (Yang et al. 2016), Gloucestershire Old Spot (Kelly et al. 2017), Iberian pigs (Crespo-Piazuelo et al. 2018), Jinhua and Landrace (Xiao et al. 2018), and Shanxi Black breeds (Gao et al. 2019). Generally, vertebrate species show distinct variations in their gut microbiota that correlate with phylogenetic changes in the host (Brooks et al. 2016; Gaulke et al. 2017; Groussin et al. 2017; Ley et al. 2008). Evidence also suggests that specific microbial signatures are heritable (Koskella et al. 2017). These close relationships suggest the co-evolution of host and gut microbiota (Brooks et al. 2016; Gaulke et al. 2017; Groussin et al. 2017; Moeller et al. 2016). The compositions and evolution of the microbiota in the digestive tracts of wild pigs have been investigated (Yang et al. 2020). In addition, host genetics may be closely involved in structuring the gut microbial communities in different species, as shown by studies in humans (Wang et al. 2021), mice (Kemis et al. 2019; Suzuki et al. 2019), and pigs (Yang et al. 2022). However, relatively little is known about the relationship between the microbial compositions and functions in specific intestinal segments and their association with the genetics and evolutionary characteristics of pigs.

The present study investigated the microbial communities in four pig breeds (Crespo-Piazuelo et al. 2018; Gao et al. 2019; Yang et al. 2020). After collection of the contents of different intestinal regions, a high-throughput sequencing analysis of the 16S rRNA gene V3–V4 region was undertaken to determine gut microbial composition variations. The phylogenetic relationships between the hosts and gut microbiota were then analyzed and functional analysis of the microbiota was conducted to assess the differences in metabolic spatial structures and pathways in the different pig breeds and how they may have influenced the phenotype and adaptation of the hosts. These results will provide the theoretical basis for the composition and evolutionary of gut microbial communities in pigs.

## Materials and methods

### Animals and sample collection

The test animals were adult female (four years old) wild pigs (WP) (Yang et al. 2020), 150-day-old Large White pigs (LW, commercial pigs), Chinese Shanxi Black pigs (CSB, indigenous pigs) (Gao et al. 2019), and 120-day-old Iberian (IB, indigenous pigs) male pigs (Crespo-Piazuelo et al. 2018). In addition, we selected three unrelated wild pigs from populations in Xingyang County in Henan Province, China. These pigs were similar genetic backgrounds and had been reared under comparable conditions. Animals raised under controlled environmental conditions and on similar diets would be expected to have less variation in their microbiota (Yang et al. 2020). The wild pigs were fed twice daily with a controlled diet consisting of corn and soybean and supplemented with hay, which would be likely to reduce variability in the microbiota relative to pigs living in the wild. The animals had free access to water, and all were healthy and had not received any antibiotic treatment (Yang et al. 2020). The LW and CSB pigs were raised individually at the Datong Pig Breeding Farm (Shanxi Province, China) on standard diets based on the feeding standard of swine (NY/T 65-2004) issued by The Ministry of Agriculture of the People’s Republic of China (Gao et al. 2019). The Iberian pigs were fed ad libitum with a standard feed containing maize, wheat, barley, and soybean, with 3320 kcal of digestible energy and 15.6% of crude protein (Crespo-Piazuelo et al. 2018). Descriptions of the processes and instructions for animal slaughter, sample collection, sample preservation, DNA extraction, library construction, and sequencing are provided in the “References” section (Crespo-Piazuelo et al. 2018; Gao et al. 2019; Yang et al. 2020). Samples were taken from four to five regions of the intestine, namely, the duodenum (DU), jejunum (JE), ileum (IL), cecum CE), and colon (CO). The wild pig’s bacterial 16S rRNA V3–V4 region was amplified using the well-documented primer pair: 338F (5′-ACTCCTACGGGAGGCAGCA-3′) and 806R (5′-GGACTACHVGGGTWTCTAAT-3′) (Yang et al. 2020). The 341F-CCTAYGGGRBGCASCAG and 806R-GGACTACNNGGGTATCTAAT primers were used to amplify the hypervariable regions (V3 and V4) of 16S rRNA genes in Shanxi Black pigs and Large White pigs (Gao et al. 2019). The Iberian pig’s bacterial 16S rRNA V3–V4 region gene was amplified with two 16 S Amplicon PCR primers: Forward, 5′TCGTCGGCAGCGTCAGATGTGTATAAGAGACAGCCTACGGGNGGCWGCAG, and Reverse, 5′GTCTCGTGGGCTCGGAGATGTGTATAAGAGACAG GACTACHVGGGTATCTAATC (Crespo-Piazuelo et al. 2018). The raw data have been uploaded to the NCBI Sequence Read Archive (SRA) database under the accession numbers PRJNA575288, SRP115844, and SRP136308. In additional, the study adhered to the guidelines on animal care of the Ministry of Science and Technology of China (Guidelines on Ethical Treatment of Experimental Animals (2006) No. 398) and the Ethics Committee of Shangqiu Normal University approved all the experiments (Shang (2022) No. 24).

### Sequence analysis

Bioinformatic analysis of the microbiomes was conducted using QIIME2 (Bolyen et al. 2018) with several slight modifications as described in the official tutorials. Briefly, the raw sequence data were demultiplexed using the “demux” plugin and cut with primers using the “cutadapt” plugin (Martin. 2011). The sequences were filtered for quality, denoised, and merged, and chimeras were removed using the “DADA2” plugin (Callahan et al. 2016). The alpha-diversity metrics Chao1 (Chao 1984), Observed species, Shannon (Shannon 1948), Simpson (Simpson 1949), Faith’s PD (Faith 1992), Pielou’s evenness (Pielou 1966), Good’s coverage (Good 1953), and beta-diversity metrics were estimated with the “diversity” plugin, and samples were rarefied to 18 321 sequences per sample. The taxonomy of the ASVs was assigned with the “classify-sklearn” naïve Bayes taxonomy classifier in the “feature-classifier” plugin (Bokulich et al. 2018) against the Greengenes Database (13.8 version; DeSantis et al. 2006).

### Bioinformatics and statistical analysis

Sequences were analyzed using QIIME2 (Bolyen et al. 2018) and several R packages (v3.2.0). The alpha diversity indices at the ASV level were determined using the ASV table in QIIME2 (Bolyen et al. 2018) and were visualized as box plots. ASV-level ranked abundance curves were created to assess richness and evenness. Jaccard (Jaccard 1908) and UniFrac (Lozupone and Knight 2005; Lozuponeet al. 2007) metrics were applied to assess structural variations in the microbiota, and the results were analyzed using PCoA and UPGMA hierarchical clustering (Ramette 2007); the significance of differentiation of microbiota structure among groups was assessed by PERMANOVA using R package “vegan.” Taxonomic differences were analyzed with MEGAN (Huson et al. 2011), GraPhlAn (Asnicar et al. 2015), and LEfSe (Segata et al. 2011). The LEfSe algorithm used a non-parametric factorial Kruskal-Wallis (KW) rank-sum test for the identification of significantly different ASVs, followed by Wilcoxon tests to examine between-group consistencies. LDA scores were used to estimate the effect sizes for differentially abundant taxa. Non-singleton ASVs were aligned using mafft (Katoh et al. 2002) and phylogenetic trees were constructed with fasttree2 (Price et al. 2009). Microbial functions were predicted by PICRUSt2 (Douglas et al. 2020) using the MetaCyc and KEGG databases.

## Results

### Data overview

Raw data analyzed was obtained from the NCBI SRA database (Crespo-Piazuelo et al. 2018; Gao et al. 2019; Yang et al. 2020). After quality control, the samples were processed using QIIME2 into 18 323 ASVs (Table S1). The presence of the ASVs was verified in the samples by species accumulation and rank-abundance curves. This showed similar patterns across the samples, indicating that the detectable microbial species were present in most samples (Fig. S1 A; Fig. S1 B).

### Diversity analysis of the pig gut microbiota

**Fig. 1:**
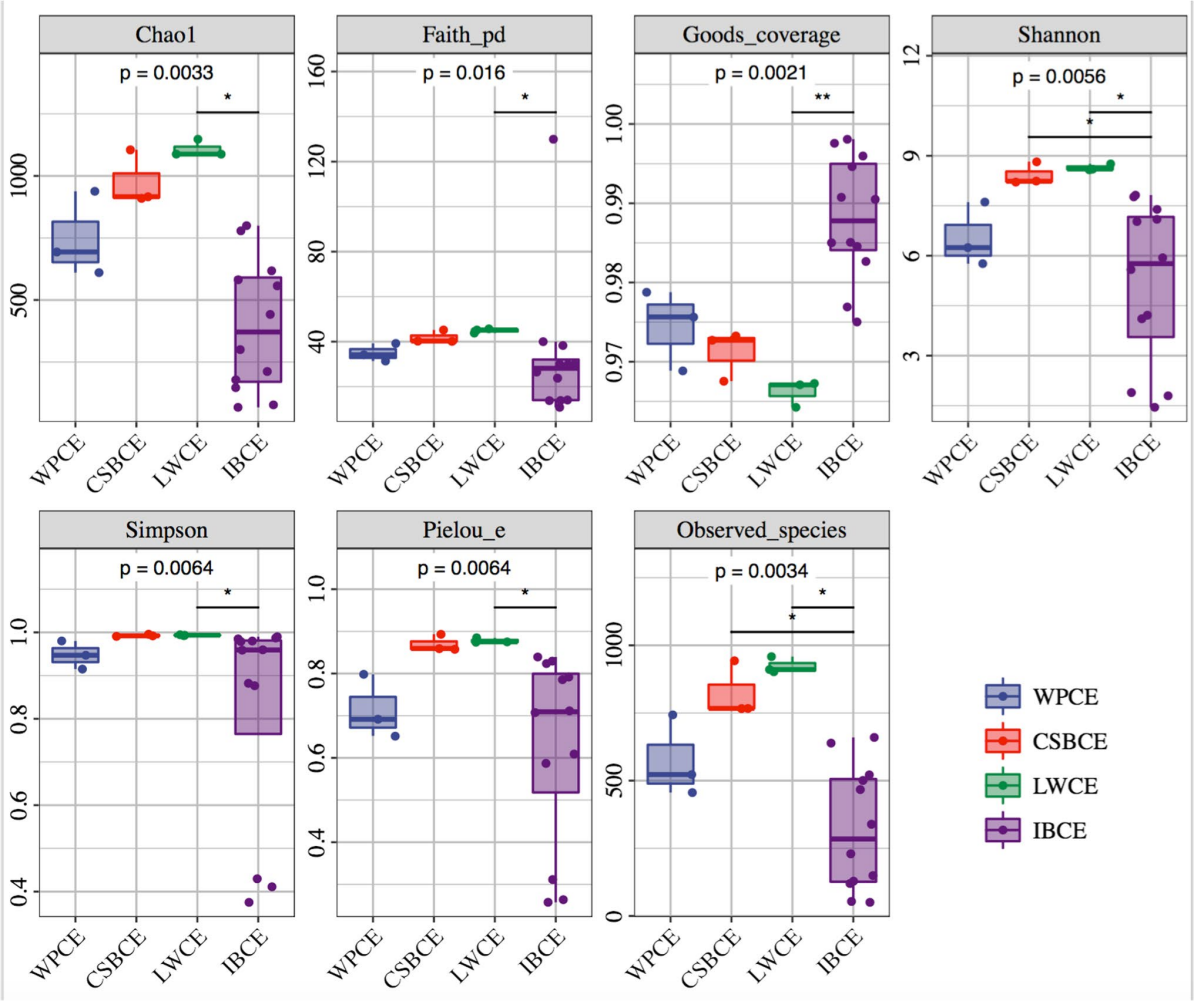
The alpha-diversity comparisons for the Wild pig cecum (WPCE), Chinese Shanxi black pig cecum (CSBCE), Large White pig cecum (LWCE), Iberian pig cecum (IBCE)

To explore the microbial composition in specific intestinal regions in the different pig breeds, we performed an initial assessment of the alpha diversity of the microbiota for each region. Significant differences in the cecal samples between the pig breeds were observed. We calculated the Chao1, Faith_PD, Simpson, and Pielou_e indices for assessment of richness and evenness. This showed that the Chao1 and Faith_PD indices were significantly reduced in IBCE samples compared with those from LWCE (*P* < 0.05; Fig. 1), while the Good’s coverage diversity index was higher in IBCE in comparison with LWCE (*P* < 0.01). No differences were seen between WP or CSB CE samples and LWCE and IBCE (*P* > 0.05; Fig. 1). The Observed species and Shannon indices were also applied for the assessment of alpha diversity to analyze species numbers and their relative abundances in the different CE samples. Both indices were observed to be significantly higher in CSBCE and LWCE samples compared with IBCE samples (*P* < 0.05; Fig. 1). Furthermore, no significant differences were seen in the Shannon and Observed species indices in the DU, JE, IL, and CO gut segments, respectively (*P* > 0.05) (Fig. S2; Fig. S3; Fig. S4; Fig. S5).

**Fig. 2:**
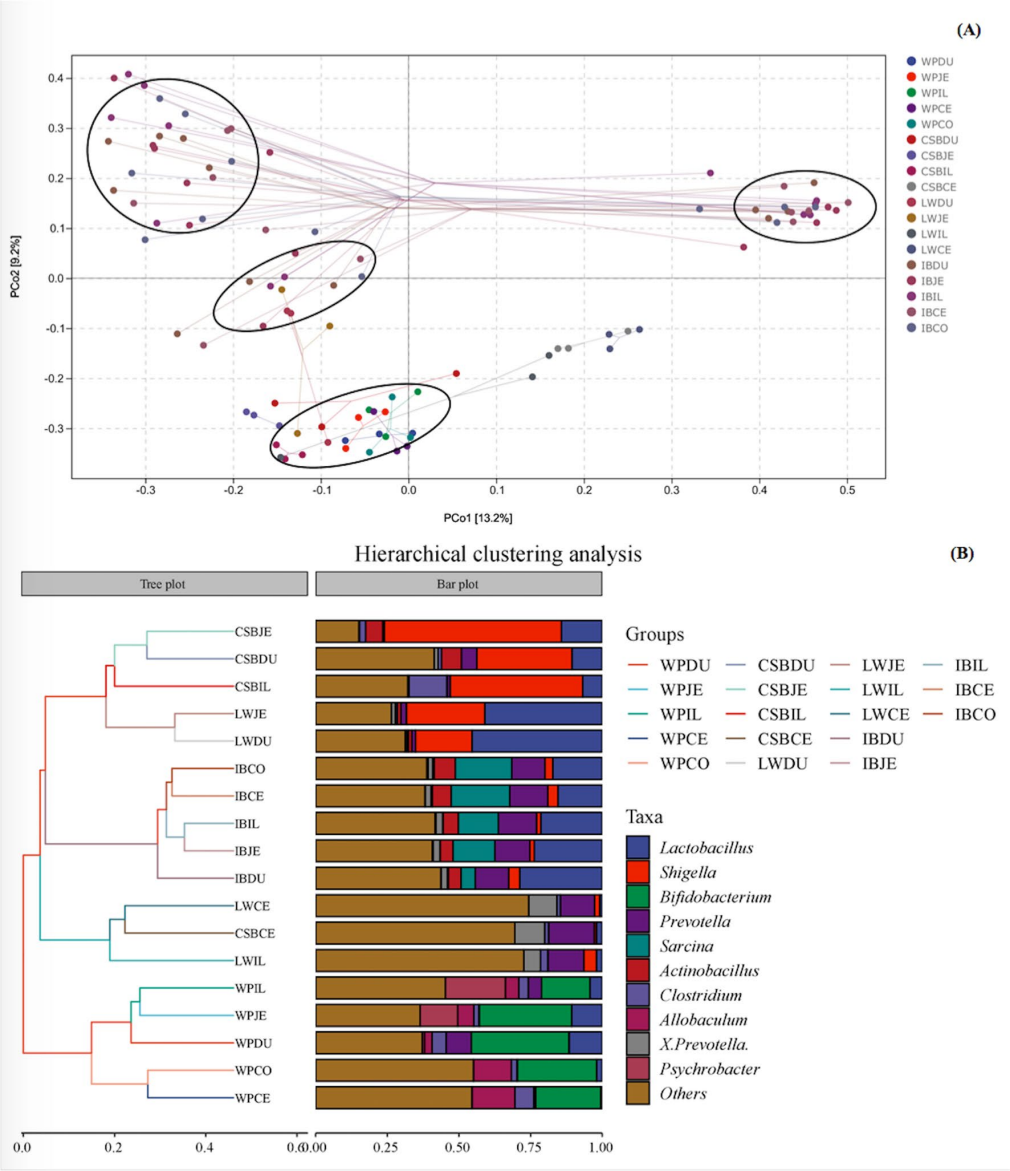
The beta-diversity comparisons for the different gut microbiota in pigs. **A** The principal coordinate analysis (PCoA) visualized via Bray-Curtis metrics and UniFrac distance metrics. Each symbol and color represents each gut location microbiota. **B** The hierarchical clustering analyses were performed by unweighted pair-group method with arithmetic means (UPGMA)

We next investigated the differences and similarities within the microbial communities of the pig breeds, using analysis of the ASVs by PCoA and UPGMA. This showed significant alterations in the gut microbiota compositions in the different intestinal regions, with the microbial composition of the DU, JE, and IL regions differing from those in the CE and CO, which resembled each other (Fig. 2A). The PERMANOVA test based on the Bray-Curtis distance measures showed that the bacterial community structure was significantly (*P* < 0.01) different among these clusters grouped (Table S2). It was further confirmed that the change in bacterial community structure was significantly correlated with pig breeds. UniFrac distance metrics clustering showed that the gut microbes tended to cluster into four groups according to the pig breed, namely, WP, CSB, LW, and IB. Additional subgroups were observed within each of the main groups, with the CE (WPCE, CSBCE, LWCE, IBCE) and CO samples (WPCO, IBCO) clustering separately from those from the IL (WPIL, CSBIL, LWIL, IBIL), DU (WPDU, CSBDU, LWDU, IBDU), and JE (WPJE, CSBJE, LWJE, IBJE) regions (Fig. 2B; Fig. S6). These findings indicated that the microbiota compositions were not uniform along the intestine in the different pig breeds, with greater similarities seen between the CE and CO samples than between samples from the DU, JE, and IL regions. As seen in Figs. 2A and B, the gut microbiota from wild pigs clustered together separately from those of both commercial and domesticated indigenous pigs.

### Microbial taxonomic composition analysis of the pig gut microbiota

**Fig. 3:**
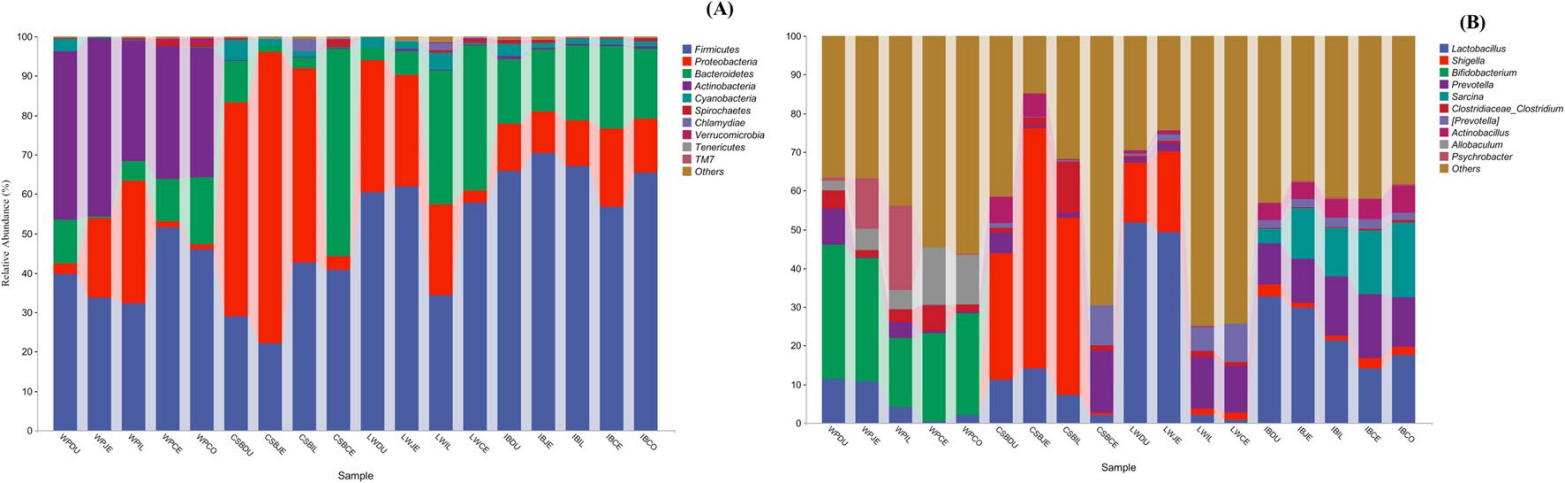
Community composition of the gut microbiota in different intestinal segments of Wild pigs, Chinese Shanxi black pigs, Large White pigs, and Iberian pigs at the phylum (**A**) and genus (**B**) levels, respectively

The taxonomic distributions of the most abundant ASVs seen in the different intestinal regions and pig breeds were then investigated. Firstly, comparing the phyla present in specific intestinal regions across the pig breeds (Fig. 3A; Table S3), it was found that *Firmicutes* were most prevalent in WP, LW, and IB, followed by *Actinobacteria* in WP, *Bacteroidetes* in IB, and *Proteobacteria* in LW. In contrast, *Proteobacteria* predominated in CSB, followed by *Firmicutes* while *Proteobacteria* levels in both CSBCE and LWCE samples were low (3.57% and 3.10%, respectively). In terms of intestinal region *Firmicutes* and *Bacteroidetes* phyla predominated and accounted for more than 80% of the bacterial phyla in IB, with *Firmicutes* and *Actinobacteria* accounting for over 80% in WP and *Firmicutes* and *Proteobacteria* accounting for over 90% in CSB and LW. Comparison of the microbial communities in samples from the same intestinal regions showed that these differed significantly with the breed of pig. For instance, the IBJE microbiota (70.38%) contained the highest abundance of *Firmicutes* than the JE of the WP, CSB, and LW (33.86%, 22.15%, 61.88%, respectively). Overall, the microbial composition differed between WP, CSB, LW, and IB for the same intestinal regions. Furthermore, we observed dynamic changes in the proportions of *Firmicutes*, *Proteobacteria*, *Bacteroidetes*, and *Actinobacteria* between the different breeds. These findings indicated that microbial communities differed between the different breeds for the same gut regions.

In terms of genera, *Bifidobacterium* and *Allobaculum* predominated in WP. In addition, *Lactobacillus*, *Prevotella*, and *Clostridiaceae* were found in DU samples from WPs, while *Shigella* and *Lactobacillus* were most abundant in CSB and LWP, and *Actinobacillus*, *Sarcina*, *Streptococcus*, *Prevotella*, *Anaerovibrio*, *Anaerovibrio*, and *Lactobacillus* predominated in IB (Fig. 3B; Table S4). In terms of distribution, it was found that only two genera (*Sarcina* and *Actinobacillus*) were absent from the five gut segments in WPs, with the lowest average distribution index of each genus seen in IL samples, suggesting that the IL microbiome is more even than that of other locations. Thus, although numerous genera were observed in the different regions of the intestine, there was a more uniform distribution in the IL (Fig. 3B; Table S4). Microbial community compositions in the same parts of the intestine also differed between wild and domesticated pigs as shown by PCoA where the domesticated pig samples were clustered and distinct from those of the wild pigs.

### Differential analysis of bacterial taxa in pig gut microbiota

**Fig. 4:**
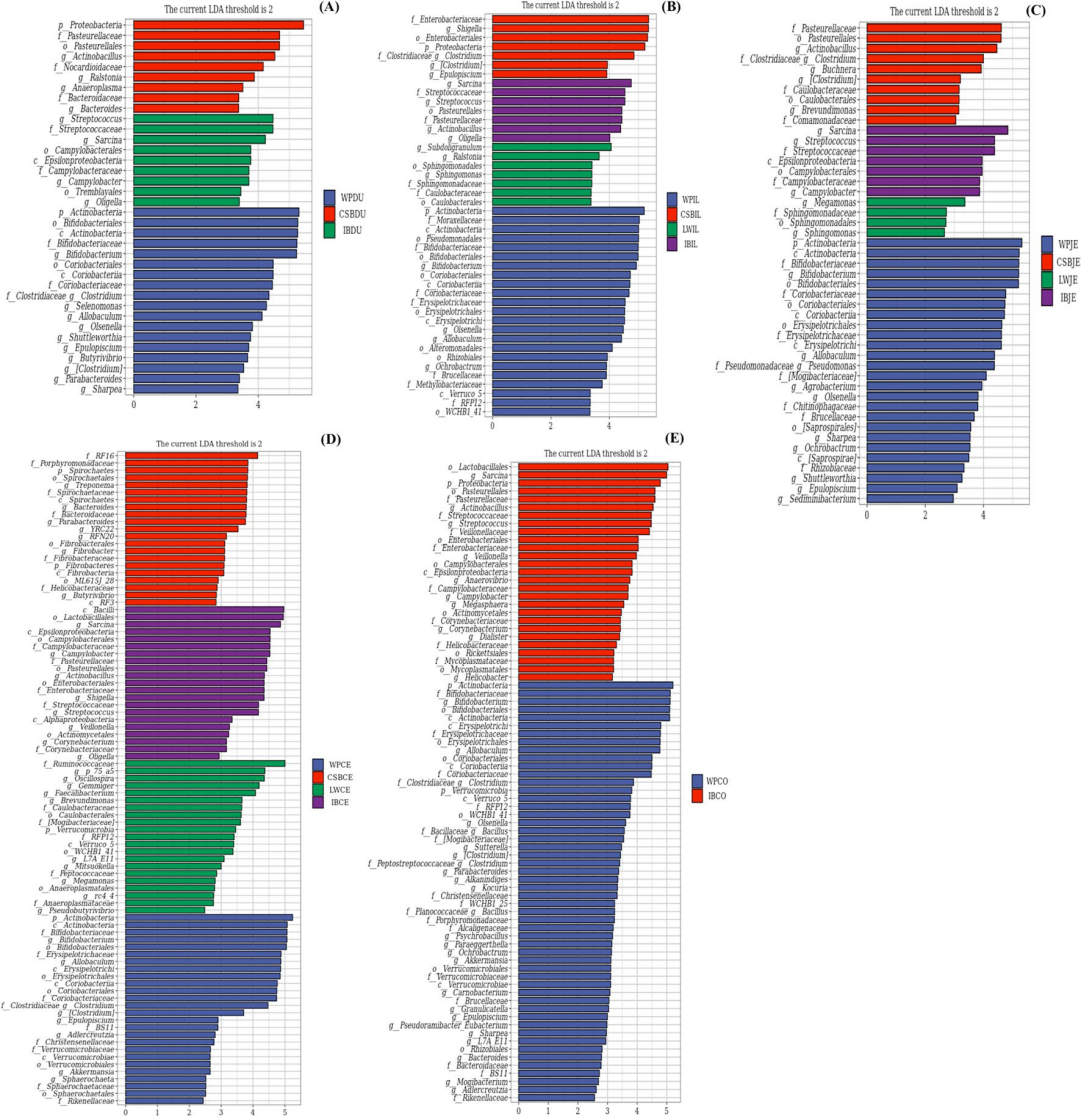
Bacterial taxa differentially represented in duodenum (**A**), ileum (**B**), jejunum (**C**), cecum (**D**), and colon (**E**) gut locations in Wild pigs, Chinese Shanxi black pigs, Large White pigs, and Iberian pigs identified by LEFSe using a LDA score threshold of > 2.0

An LEfSe analysis was performed to determine the microbial species characteristics of different intestinal regions. Using LDA scores > 2, this showed differential abundances of 36, 47, 44, 89, and 79 ASVs in DU, JE, IL, CE, and CO samples, respectively, in the WP, CSB, LW, and IB groups (Fig. 4; Table S5). Differences were observed between the microbiomes of WPs and those of the LW, CBS, and IB pig breeds, seen at all taxonomic levels from phylum to genus (Fig. 4; Table S5). As seen in Fig. 4A, WPDU showed a significantly greater abundance in 17 taxa in *Firmicutes*, nine taxa in *Actinobacteria*, and five taxa each in *Bacteroidetes* and *Proteobacteria*. In comparison, IBDU samples contained greater abundances of 10 taxa in *Proteobacteria*, 11 in *Firmicutes*, and five taxa in *Actinobacteria*, while CSBDU showed had nine taxa in *Proteobacteria*, five taxa each in *Bacteroidetes* and *Tenericutes*, and four taxa in *Actinobacteria.* Thus, at the phylum level, *Actinobacteria* represented a significant biomarker for the various intestinal segments in WPs compared with IB, CSB, and LW. Similarly, *Proteobacteria* represented a biomarker for the DU and IL regions in CSB, in contrast to IB, WP, and LW, and was also a biomarker for IBCO compared with WPCO. At the genus level, it was found that *Bifidobacterium* and *Allobaculum* showed the greatest differences in abundance in the different intestinal regions of WPs compared with IB, WP, and LW. These differences were maintained in the order, class, and family levels (Fig. 4; Table S5). In addition, *Sarcina* and *Streptococcus* were more abundant throughout the gut of IB pigs, compared with CSB, WP, and LW, while *Actinobacteria* was more abundant in the DU and JE regions in CSB. WP samples showed enrichment of both *Bifidobacterium* and *Allobaculum* in comparison with domesticated pigs, while the latter showed a greater abundance of *Shigella* and *Lactobacillus* in CSB and LW and *Actinobacillus* and *Sarcina* in IB. These findings indicate that the domesticated pig microbiota has diverged from its ancestral state.

### Phylogenetic analysis of the pig gut microbiota

**Fig. 5:**
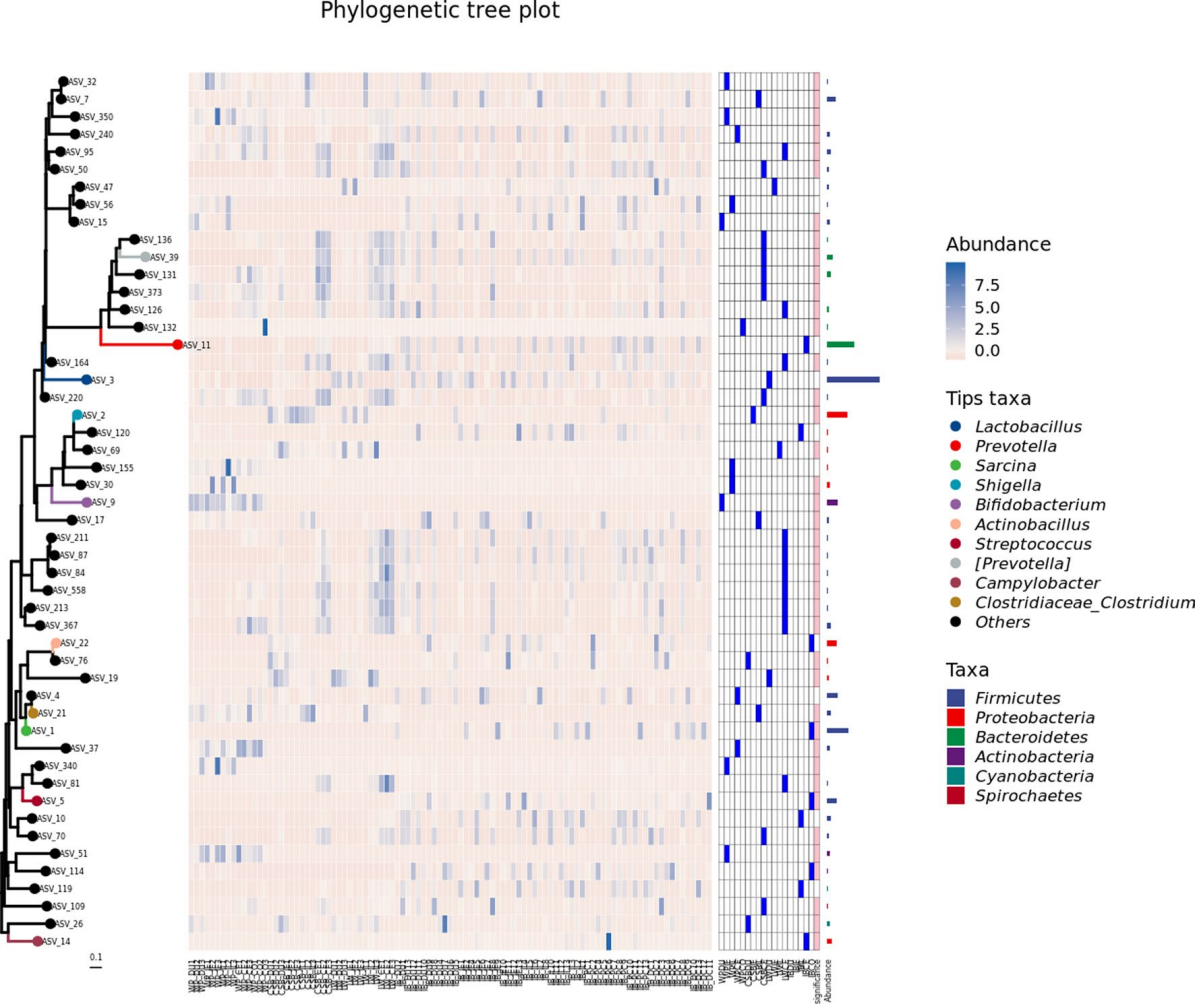
Phylogenetic tree with ASV abundance distribution. Species abundance distribution was aligned to the tree and visualized as boxplots. The phylum information was used to color symbolic points on the tree and also species abundance distributions

A phylogenetic tree of the microbial ASVs was created to investigate the evolutionary relationships between the gut microbiota from the different pig breeds, using distance matrices calculated from the sequencing analysis. The tree was generated using the most abundant 50 bacterial genomes represented in the pig microbiome (Fig. 5; Fig. S7; Fig. S8; Fig. S9; Fig. S10; Fig. S11). This showed that the genomes spanned many microbiota-associated phyla. Each branch of the tree represents one gut microbiota. Phylogenetic trees for *[Prevotella]* (ASV39), *Prevotella* (ASV11), *Lactobacillus* (ASV3), *Shigella* (ASV2), *Bifidobacterium* (ASV9), *Actinobacillus* (ASV22), *Clostridium* (ASV21), *Sarcina* (ASV1), *Streptococcus* (ASV5), and *Campylobacter* (ASV14) showed that their microbiomes represent “core” microbial genera that do not cluster together. Interestingly, the top 50 most abundant microbiota were located on different branches according to their intestinal location with related organisms clustered together. Several clusters of co-existing bacteria were identified by visual inspection, with the 10 most abundant genera indicated in Fig. 5. The different clusters tended to be associated with pig breeds, for example, clusters 1 (ASV_32, ASV_7, ASV_350, ASV_240, ASV_95, ASV_50, ASV_47, ASV_56, ASV_15, ASV_164), 2 (ASV_136, ASV_39, ASV_131, ASV_373, ASV_126, ASV_132, ASV_11), and 3 (ASV_2, ASV_120, ASV_69, ASV_155, ASV_30) are associated with different phyla, *Firmicutes*, *Bacteroidetes,* and *Proteobacteria* in different pig breeds. This relationship with the host type is suggestive of specific interactions between the microbiota and the host. Cluster 1, for example, while consisting largely of *Firmicutes*, also includes other phyla, while in cluster 3, the constituents are present in all the breeds but belong to the same microbial group. Thus, phylogeny is able to demonstrate the evolution of microbiota during the domestication of pig.

### Functional analysis of the pig gut microbiota

**Fig. 6:**
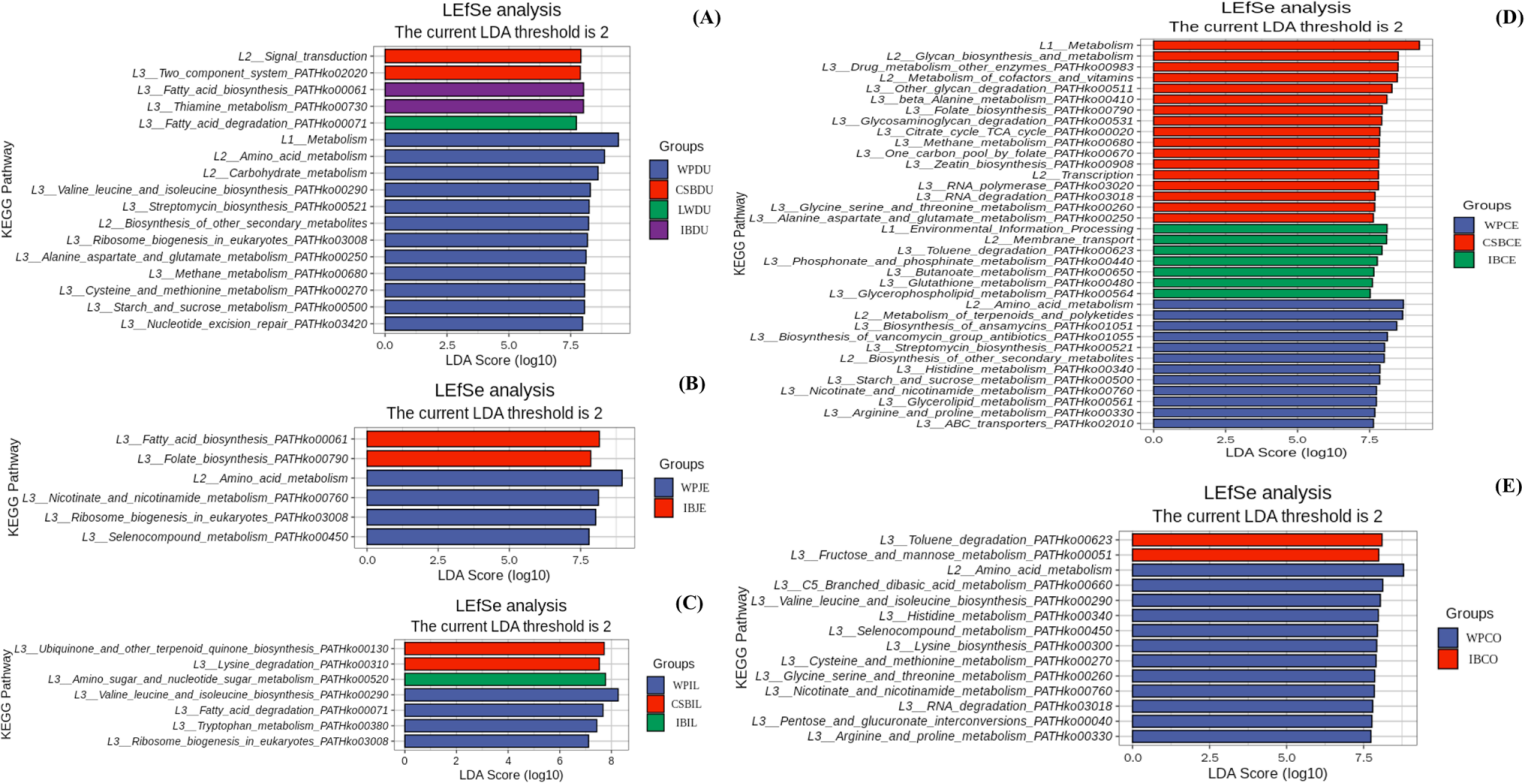
Predicted functional differentially of the bacterial genus represented in the duodenum (**A**), jejunum (**B**), ileum (**C**), cecum (**D**), and colon (**E**) gut locations in different pig breeds identified by LEFSe using a LDA score threshold of > 2.0

**Fig. 7:**
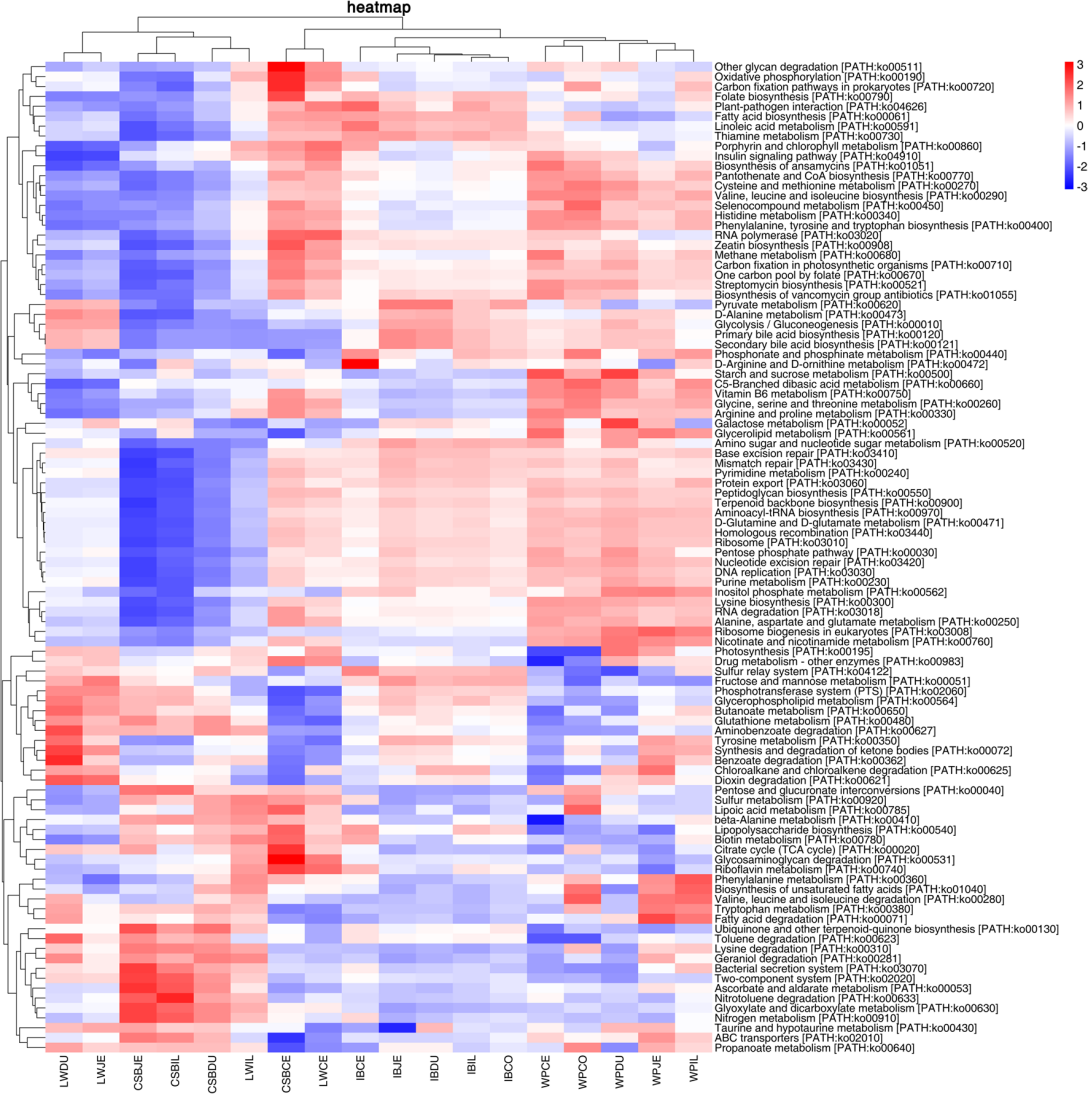
Heatmap clustered by KEGG pathway showing different enrichments in the different gastrointestinal sites and different pig breeds. The vertical columns represent groups, and the horizontal rows depict metabolic pathways. The color coding is based on row *z*-scores

PICRUSt2 was used for metagenomic assessment of the identified bacteria, as this may provide insight into metabolic differences in the various intestinal regions of the host. The ASVs were assigned to genes using available genetic annotations, and the functions of the genes were analyzed by KEGG pathway enrichment. This yielded 7746 genes (Table S6) associated with 393 pathways (Table S7). Seven pathways were observed to differ between the different intestinal regions. The most enriched functional modules in all samples were “human diseases,” “organismal systems,” and “metabolism,” while the most significant pathways were also similar in the different samples. Six of these pathways were related to metabolism (amino acid, biosynthesis, carbohydrate, energy, cofactors and vitamins, and lipid), with one pathway involved in the processing of environmental information (member transport) and two pathways associated with the processing of genetic information (translation and replication, and repair) which were common to all samples (Table S7). The associations of these pathways with the same intestinal samples from different pig breeds were then examined. As seen in Fig. 6, the pathway enrichment differed according to the intestinal region in the different breeds with 12 pathways showing significant enrichment in the DU, five in the JE, seven in the IL, 26 in the CE, and 13 in the CO (*P* < 0.05; Fig. 6; Table S8). The enriched pathways in the DU were “fatty acid degradation” (ko00071) in LWDU and “fatty acid biosynthesis” (ko00061) in IBDU. The DU also had a greater abundance of microbial genes in the “methane metabolism” (ko00680) and “starch and sucrose metabolism” (ko00500) in WPDU. The pathways enriched in JE and IL samples were “lysine degradation” (ko00310), “ubiquinone and other terpenoid quinone biosynthesis” (ko00130) in CSBIL, “fatty acid biosynthesis” (ko00061), “folate biosynthesis” (ko00790), and “amino sugar and nucleotide sugar metabolism” (ko00520) in IBJE and IBIL, “nicotinate and nicotinamide metabolism” (ko00760), “seleno-compound metabolism” (ko00450), “ribosome biogenesis in eukaryotes” (ko03008), “fatty acid degradation” (ko00071), “valine, leucine, and isoleucine biosynthesis” (ko00290), and “tryptophan metabolism” (ko00380) in WPJE and WPIL. Furthermore, it was found that CSBCE samples had highest abundance of microbial genes in “zeatin biosynthesis” (ko00908), “drug metabolism other enzymes” (ko00983), “other glycan degradation” (ko00511), “glycosaminoglycan degradation” (ko00531), “citrate cycle TCA cycle” (ko00020), “RNA degradation” (ko03018), “beta alanine metabolism” (ko00410), “folate biosynthesis” (ko00790), “RNA polymerase” (ko03020), and “methane metabolism” (ko00680) in CSB compared with WPCE (starch and sucrose metabolism (ko00500), biosynthesis of ansamycins (ko01051), streptomycin biosynthesis (ko00521), histidine metabolism (ko00340), biosynthesis of vancomycin group antibiotics (ko01055), ABC transporters (ko02010) and IBCE (phosphonate and phosphinate metabolism (ko00440), toluene degradation (ko00623), glycerophospholipid metabolism (ko00564), and butanoate metabolism (ko00650) in the WP and IB breeds. The IBCO microbial genes were related to “fructose and mannose metabolism” (ko00051) and “toluene degradation” (ko00623) which had low abundance in the IB samples. In contract, WPCO samples showed greater enrichment in “nicotinate and nicotinamide metabolism” (ko00760), “seleno-compound metabolism” (ko00450), “pentose and glucuronate interconversions” (ko00040), “RNA degradation” (ko03018), “histidine metabolism” (ko00340), and “lysine biosynthesis” (ko00300). The clustered heatmap showed a clear distinction between the WP and IB samples and the CSB and LW samples (Fig. 7; Table S9). Notably, more microbial genes associated with metabolism were found in wild pigs. However, the relative abundance of microbial genes related to the metabolism of amino acids and carbohydrates, as well as resistance, was greater in WPs in comparison with the LW, CSB, and IB domesticated pigs, while genes associated with lipid metabolism were more abundant in IB pigs compared with LW, CSB, and WP pigs. These results suggest that the metabolic functions of the microbiota differ according to pig breeds, with specific pathways predominating in certain breeds.

## Discussion

The literature contains relatively few studies on the microbial composition and potential functionality of the fecal microbiota in wild pigs, domestic indigenous pigs, and commercial pigs (Ushida et al. 2016; Huang et al. 2020). The relationships between specific microbial composition and evolution have been characterized in wild pigs (Yang et al. 2020). An analysis of the gut microbiota in the gastrointestinal tracts of wild boar in the karst region of Southwest China also demonstrated the importance of environmental adaption (Cao et al. 2022). To date, few studies have specifically focused on the effect of domestication on the evolutionary of the gut microbiota in pigs, and it has been assumed that domestication has resulted in only minor changes in community richness. Furthermore, the key to understanding the role of disrupted microbiota in pigs is to specifically consider the composition and evolution of the microbiota themselves and whether inter-group microbial differences can be linked to domestication. We, therefore, systematically characterized changes in the microbial composition, distribution, phylogeny, and potential functionality in specific sections of the digestive tract in different pig breeds. This showed vast differences in microbial composition along the length of the gut, and we, furthermore, provide additional insights into the composition and evolutionary of the gut microbiota in each section and their potential functional consequences in pigs.

At the phylum level, the primary findings centered on the structural composition and distribution of the intestinal microbiota in WP, LW, CSB, and IB pig breeds (Fig. 3A; Table S3). Earlier work has observed the core microbiota of domestic pigs to be dominated by *Firmicutes* and *Bacteroidetes* (Crespo-Piazuelo et al. 2018; Gao et al. 2019; Xiao et al. 2018; Yang et al. 2016; Mach et al. 2015; Ramayo-Caldas et al. 2016; Quan et al. 2018, 2019; He et al. 2016; Ivarsson et al. 2014; Kraler et al. 2016; Liu et al. 2012; Slifierz et al. 2015). Here, we observed that the four most abundant phyla were *Firmicutes, Proteobacteria*, *Bacteroidetes*, and *Actinobacteria*. The *Bifidobacterium* and *Allobaculum* genera predominated in WPs, while *Shigella* and *Lactobacillus* genera were most abundant in CSB and LWP and *Actinobacillus* and *Sarcina* in IB pigs. In contrast, neither *Sarcina* and *Actinobacillus* were found in any of the gut regions of WPs (Fig. 3B; Table S4). These findings indicate that many bacterial species are uniformly distributed throughout the intestine in same pig breeds. Previous studies have reported marked differences in pig microbiota at the genus level. For instance, *Lactobacillus*, *Prevotella*, and *Treponema* were predominant in Duroc pigs (Yang et al. 2016), while Xiao et al. (2017) observed a greater abundance of *Prevotella*, *Clostridium*, *SMB53*, and *Streptococcus* in Landrace, and Yorkshire and Hampshire pigs. These differences may result from differences in feed composition and environment, as well as from inherent breed differences. However, it is difficult to make detailed comparisons of the specific effects of diet and environment in the studied populations.

Multiple factors are known to affect the gut microbiota, including genetics. Human genetic variation is documented to influence the gut microbiota by both environmental and host-associated factors (Bonder et al. 2016; Qin et al. 2022; Lopera-Maya et al. 2022). Genetic variations result in phenotypic differences, which are also known to influence the gut microbiota (Kemis et al. 2019; Suzuki et al. 2019; Chen et al. 2020; Wang et al. 2021; Yang et al. 2022). Chen et al. (2021a) determined the stability and variation in human microbiota in response to physiological changes in the host. Yang et al. (2022) have shown that, under conditions of significant genetic diversity and environmental uniformity, the composition of the microbiota is heritable. These results provide strong evidence for the influence of host genotype on the abundance of specific bacteria in the intestine. Thus, the pig genotype can affect the composition of the gut microbiota. Differences at the levels of phylum, class, order, family, and genus were apparent in samples from the same intestinal region in different pig breeds (Fig. 4; Table S5). Within-group stability was observed for both microbial composition and abundance. We also found that the genetic stability of the microbial communities varied significantly according to pig breed, with *Bifidobacterium* species showing relatively high within-group stability over extended periods in WP populations. Notably, it has been found that some *Bifidobacterium* species colonize wild pig populations and subsequently show a high degree of genetic stability (Ushida et al. 2016). This supports the observation that the microbiota of both indigenous and commercial pigs have diversified from their state in ancestral pig populations. Martínez and colleagues (2018) reported that each person has a unique and stable community of gut microbes that is as personal as a “fingerprint.” Studies have shown that an individual’s genetics, diet, environment, lifestyle, and physiological state all make small contributions to variations in the gut microbiome among individuals. However, less than 30% of this variation can be explained, and even identical twins, who share the same genotype and often diets and lifestyle, have distinct gut microbiomes. It is interesting that, despite living in varied habitats, wild pigs show remarkable similarities in their microbiota. More importantly, the microbiota of domesticated pigs differed significantly from those of their wild ancestors. These findings also suggest that different pig breeds may have specific microbial compositions that differ from others and may persist for long periods of time. The genetic profile of the gut microbiota may thus represent a host “marker” that distinguishes the specific host from others. Overall, these results demonstrate that both the composition and functional influence of the microbiota may be closely linked with the genotype of the host. Gut microbes are vertically transmitted in pigs, and thus, there is a symbiotic relationship between the hosts and their associated microbial communities. Therefore, it is not surprising that the effects of these factors were greater in modern pig breeds. The aim of the study was not to demonstrate that domestication and the development of the modern breeds are the most critical factors affecting the pig gut microbiota. Instead, we investigated and confirmed that pig domestication resulted in a specific shift in the composition of the pig microbiome. Moreover, we argue that the transition from ancestral wild pigs to modern pig breeds is masked to some extent by the influence of genotype, which may independently drive changes in the microbiome.

Phylogenetic analysis showed that pig genotypes were associated with a variety of microbiota phyla (Fig. 5). Each branch on the tree represents one gut microbiota. Phylogenetic trees for *Prevotella* (ASV11), *Lactobacillus* (ASV3), *Shigella* (ASV2), *Bifidobacterium* (ASV9), *Sarcina* (ASV1), and *Streptococcus* (ASV5) showed the presence of “core” microbial species in different groups. Interestingly, the phylogenetic trees showed that the top 50 most abundant gut microbes from different intestinal locations were located on different branches in different pig populations and were clustered according to the taxonomy of the microorganisms. Several clusters of bacteria were visible after clustering, as shown in Fig. 5. These bacteria belonged to a variety of species, usually belonging to the same order. PCoA and UPGMA analyses showed a lack of uniformity in the gut microbiota along the intestine among the different pig breeds, while within the same breed, there tended to be clustering of the microbiota species. Specifically, the gut microbial clusters from WPs were distinct from those of the domesticated CSB, IB, and LW (Fig. 3A and B). Sharpton has described mechanisms through which gut microbes in vertebrates affect the physiology and fitness of the hosts (Sharpton 2018). Wibowo et al. (2021) performed a large-scale de novo assembly of microbial genomes from palaeofeces, establishing that palaeofeces with well-preserved DNA are abundant sources of microbial genomes, including previously undescribed microbial species, which may elucidate the evolutionary histories of human microbiomes. The phylogenetic analysis showed significant correspondences between the gut microbes and their hosts, strongly suggestive of co-evolution. This also suggests that the adaptation is to the host genotype, as this had a significant effect on the composition of the microbial communities. Thus, phylogenetic analysis can be used to investigate the evolution of microbiota during the change from wild to domesticated conditions. In all, the findings suggest that the composition and functional influence of the microbiota are closely linked with the evolutionary adaptation of the host, suggestive of the co-evolution of pigs and their microbiota.

In the final section, we performed a metagenomic analysis of the functional influence of the microbiota in the different pig breeds. A total of 7746 genes (Table S6) associated with 393 pathways (Table S7) were identified by KEGG. As shown in Fig. 6, the five intestinal regions differed in the functions of their microbiota among the four pig populations (*P* < 0.05; Fig. 6; Table S8). The clustered heatmap shows a clear distinction between WP and IB samples and CSB and LW samples (Fig. 7; Table S8). Specifically, the microbiota from WPs contained greater numbers of metabolism-related genes. Different abundances were seen in approximately half of the bacterial species and pathways, together with within-group differences in microbial genotypes. Genes associated with amino acid and carbohydrate metabolism, as well as resistance, were more abundant in WPs compared with LW, CSB, and IB, while genes associated with lipid metabolism were more common in IB pigs than in LW, CSB, and WP pigs. These four types of pigs differ in their lipid content: CSB and IB pigs have higher fat deposition with lower proportions of meat compared with the leaner LW and WP pigs. Gao et al. (2019) observed that *Prevotella* was more abundant in CSB pigs compared with their LW counterparts, suggesting that CSB pigs may be more efficient in their absorption of nutrients than LW pigs. Crespo-Piazuelo et al. (2018) reported that energy pathways associated with the gut microbiota varied along the intestine in IB pigs, suggesting an explanation for the influence of the microbiota on lipid metabolism. Anaerobic bacteria compete for the degradation of plant polysaccharides and fatty acid production in the colon. *Prevotella* is usually abundant in pig feces (Yang et al. 2016; Kim et al. 2017) and is known to be involved in polysaccharide degradation and amino acid metabolism to influence the level of intramuscular fat in pigs (Fang et al. 2017). Chen et al. (2021b) suggested that *Prevotella copri* in the intestines of pigs fed on commercial diets influences chronic inflammatory responses in the host, mediated by TLR4 and mTOR signaling to increase fat deposition in the host. Thus, differences in the lipid content of the host may be caused by microbes such as *Prevotella* spp. Further investigation is necessary to verify these possible relationships. Furthermore, we observed that *Actinobacteria* predominated in the intestines of WPs, whereas *Bacteroidetes* were more abundant in domesticated pigs. *Bacteroidetes* are able to degrade bacterial exopolysaccharides in the animal gut (Lammerts van Bueren et al. 2015), while *Actinobacteria* produce various compounds that influence immunity and metabolism and are vital to host health (Matsui et al. 2012). The change from dominance by *Actinobacteria* in wild pigs to *Bacteroidetes* in domesticated pigs may be the result of a variety of influences, including genetics, evolution, environmental change, and the type of food. *Actinobacteria* are also associated with resistance against infection and higher levels of this phylum in wild pigs may be associated with increased infection resistance in contrast to domesticated pigs. Thus, it is apparent that domestication has resulted in a shift in the composition of the microbiota, and although there are clear similarities between wild and domesticated animals in terms of microbial dominance, there are clear differences between the two populations.

The *Bifidobacterium* and *Allobaculum* genera predominated in most of the WP samples (Fig. 3B; Table S4). Bifidobacteria are found in the digestive tracts of both mammals and insects (Ventura et al. 2012) and, together with *Allobaculum*, are classified as probiotic bacteria as they protect the mucosal barrier of the intestine (Furusawa et al. 2013). These bacteria contain numerous genes encoding enzymes responsible for the breakdown of complex carbohydrates (Milani et al. 2014). *Bifidobacterium* degrade the hexose sugars through a specific pathway, the “bifid shunt.” dependent on the fructose-6-phosphate phosphoketolase enzyme (Pokusaeva et al. 2011). The end-products of this pathway are ATP and short-chain fatty acids that protect the mucosal barrier against pathogenic infection (Sánchez et al. 2010); an example is acetate produced by *Bifidobacterium* which has been shown to protect epithelial cells from infection (Fukuda et al. 2011). *Bifidobacterium* has also been found to protect against rotaviral enteritis (Rigo-Adrover et al. 2017) and necrotizing enterocolitis in newborn rats (Satoh et al. 2016; Wu et al. 2013) and to enhance inflammation and the immune response in colitis-affected rats during weaning (Izumi et al. 2015). Similarly, one of the most abundant genera observed in wild pigs was *Allobaculum*, a member of the *Erysipelotrichaceae* family and about which relatively little is known. The first reported member of the genus is *Allobaculum stercoricanis* from dog feces (Greetham et al. 2004). *Allobaculum* is also negatively associated with adiposity in mice (Baldwin et al. 2016) and has been found to utilize glucose and produce both lactate and butyrate (Herrmann et al. 2017). *Allobaculum* species have also been linked to inflammation in mice (Cox et al. 2014), while Miyauchi et al. (2020) observed a causative association between an *Allobaculum* strain and a predisposition to autoimmune encephalitis. Van Muijlwijk et al. (2021) observed that *A. mucolyticum* secreted numerous O-glycan carbohydrate-degrading enzymes, resulting in an ability to degrade intestinal mucins, resulting in effective colonization and degradation of the intestinal mucus layer. Thus, it is possible that the high proportion of *Bifidobacterium* and *Allobaculum* in the microbiome may enhance both nutrient absorption and resistance to disease in wild pigs. However, Tsuchida et al. (2017) observed that intestinal bacteria from wild pigs contained fiber-degrading enzymes while domestic pigs expressed genes associated with tetracycline resistance. It is possible that the latter may be linked to the routine use of antibiotics during the rearing of domestic pigs. Overall, these reports suggest that throughout porcine evolution and domestication, alterations in both the environment and nutrient source, as well as artificial selection, have led to divergence in the composition of the intestinal microbiome, with modern domestic pigs having microbiota associated with fast growth but reduced disease resistance compared with wild pigs (Yang et al. 2020). The metagenomic results suggest that the microbiota have distinct spatial and functional attributes that enhance the degradation and utilization of nutrients as well as maintaining gut homeostasis. We have also demonstrated a possible causal association between microbial, metabolites, and the host phenotype. There is the potential to expand knowledge of how physical damage to the gastrointestinal tract alters microbial content. An environmental application of the pig gastrointestinal model could evaluate the effects of climate change on the microbiome and gut health (Lunney et al. 2021). Our future studies will focus on the microbial strain, investigating genetics and evolution by metagenomic sequencing or whole genome sequencing, as well as the potential therapeutic effects of diet on the gut microbiome.

## Conclusion

Significant differences in microbial compositions and functions were found in the same intestinal regions of different pig breeds. The results suggest that the evolution of these aspects of the microbiota is strongly linked to both the domestication and genetics of the pig. Microbiota were found to be genetically stable and specific, with vertical transmission in the host, suggesting co-evolution of the host and its microbiome. The gut microbiota influences the host, with signaling pathways affecting the phenotype of the host. Nevertheless, the influence of the highly variable microbial genomes on the metabolism of the host remains to be explored. These findings provide insight into the complexity of microbial-host relationships and highlight the significance of applying this knowledge in agricultural practice.

### Supplementary information

Below is the link to the electronic supplementary material.

Supplementary file1 (PNG 418 KB) Fig. S1 Species accumulation (A) and rank-abundance (B) curves analysis of the different gut intestinal tract samples at 97% sequences identity. If the curves reach or nearly reach a plateau, it indicates that most of the species present in all samples have been observed.

Supplementary file2 (PNG 78 KB) Fig. S2 The alpha-diversity comparisons for the duodenum (DU) in different pig populations.

Supplementary file3 (PNG 71 KB) Fig. S3 The alpha-diversity comparisons for the jejunum (JE) in different pig populations.

Supplementary file4 (PNG 70 KB) Fig. S4 The alpha-diversity comparisons for the ileum (IL) in different pig populations.

Supplementary file5 (PNG 57 KB) Fig. S5 The alpha-diversity comparisons for the colon (CO) in different pig populations.

Supplementary file6 (PNG 1499 KB) Fig. S6 The hierarchical clustering analyses were performed by unweighted pair-group method with arithmetic means (UPGMA).

Supplementary file7 (PNG 115 KB) Fig. S7 Phylogenetic tree with ASV abundance distribution in duodenum (DU) of pigs. Species abundance distribution was aligned to the tree and visualized as boxplots. The Phylum information was used to color symbolic points on the tree and also species abundance distributions.

Supplementary file8 (PNG 110 KB) Fig. S8 Phylogenetic tree with ASV abundance distribution in jejunum (JE) of pigs. Species abundance distribution was aligned to the tree and visualized as boxplots. The Phylum information was used to color symbolic points on the tree and also species abundance distributions.

Supplementary file9 (PNG 118 KB) Fig. S9 Phylogenetic tree with ASV abundance distribution in ileum (IL) of pigs. Species abundance distribution was aligned to the tree and visualized as boxplots. The Phylum information was used to color symbolic points on the tree and also species abundance distributions.

Supplementary file10 (PNG 116 KB) Fig. S10 Phylogenetic tree with ASV abundance distribution in cecum (CE) of pigs. Species abundance distribution was aligned to the tree and visualized as boxplots. The Phylum information was used to color symbolic points on the tree and also species abundance distributions.

Supplementary file11 (PNG 108 KB) Fig. S11 Phylogenetic tree with ASV abundance distribution in colon (CO) of pigs. Species abundance distribution was aligned to the tree and visualized as boxplots. The Phylum information was used to color symbolic points on the tree and also species abundance distributions.

Supplementary file12 (XLSX 5870 KB)

Supplementary file13 (XLSX 36 KB)

Supplementary file14 (XLSX 53 KB)

Supplementary file15 (XLSX 46 KB)

Supplementary file16 (XLSX 63 KB)

Supplementary file17 (XLSX 6261 KB)

Supplementary file18 (XLSX 350 KB)

Supplementary file19 (XLSX 51 KB)

Supplementary file20 (XLSX 119 KB)

## Data Availability

All data generated during and/or analyzed during the current study are available from the corresponding author on reasonable request.
