# Supplementary figures and images for: Composition and evolutionary characterization of the gut microbiota in pigs
Source: Int Microbiol. 2023 Nov 20;27(4):993–1008. doi: 10.1007/s10123-023-00449-8 (PMC11300507; doi:10.1007/s10123-023-00449-8)

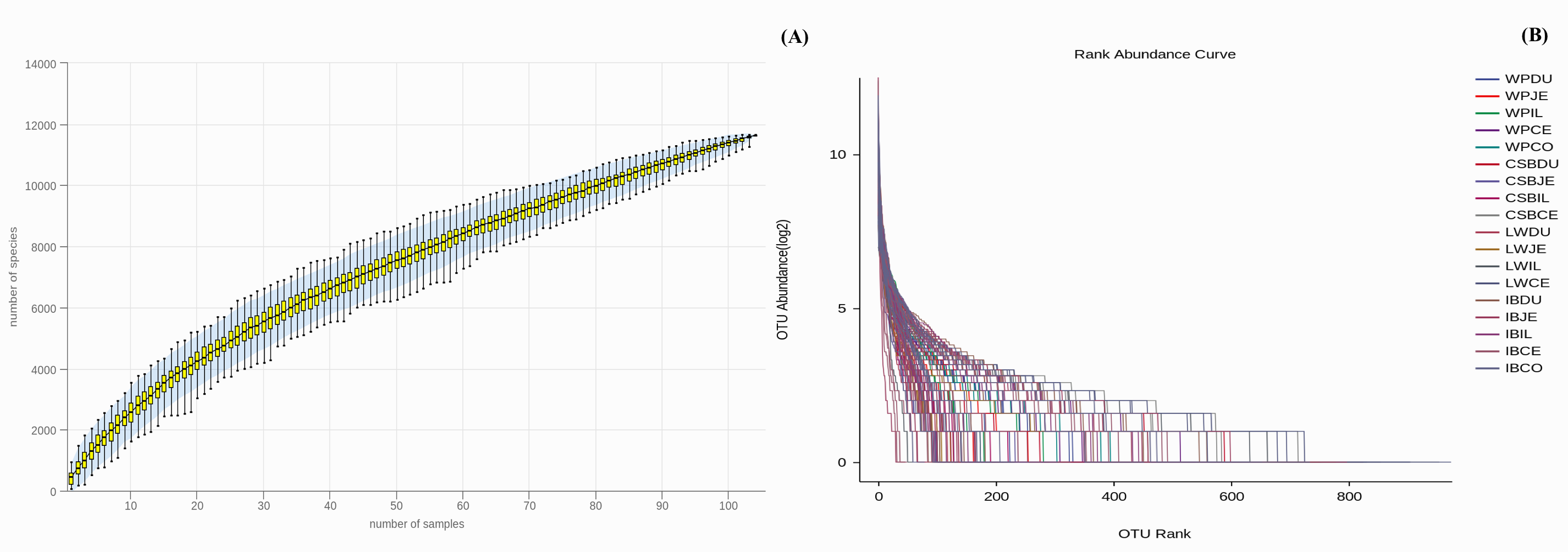

Supplement: Supplementary file 1 — Supplementary file1 (PNG 418 KB) Fig. S1 Species accumulation (A) and rank-abundance (B) curves analysis of the different gut intestinal tract samples at 97% sequences identity. If the curves reach or nearly reach a plateau, it indicates that most of the species present in all samples have been observed. [file 10123_2023_449_MOESM1_ESM.png]

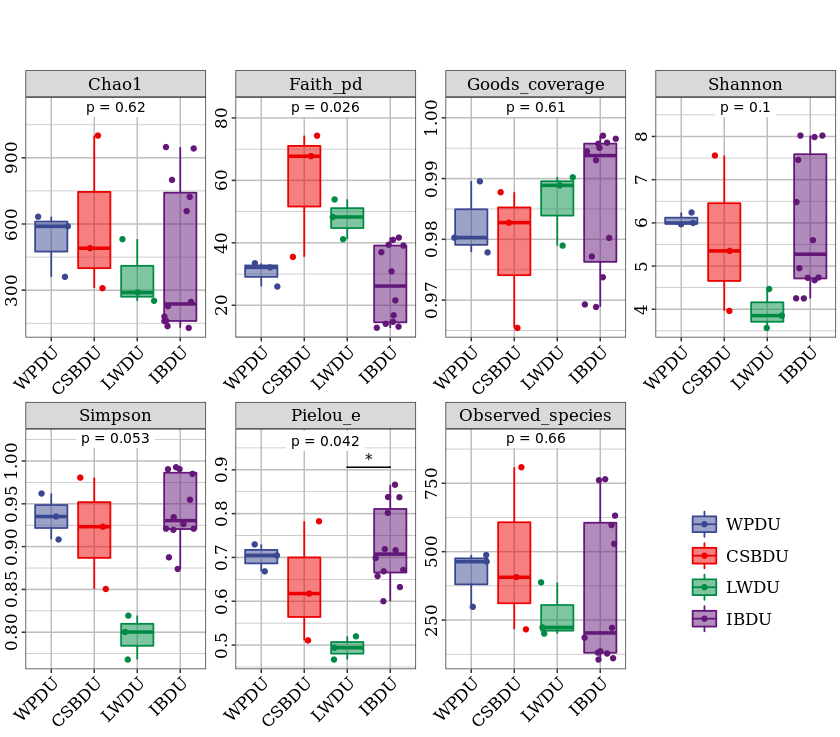

Supplement: Supplementary file 2 — Supplementary file2 (PNG 78 KB) Fig. S2 The alpha-diversity comparisons for the duodenum (DU) in different pig populations. [file 10123_2023_449_MOESM2_ESM.png]

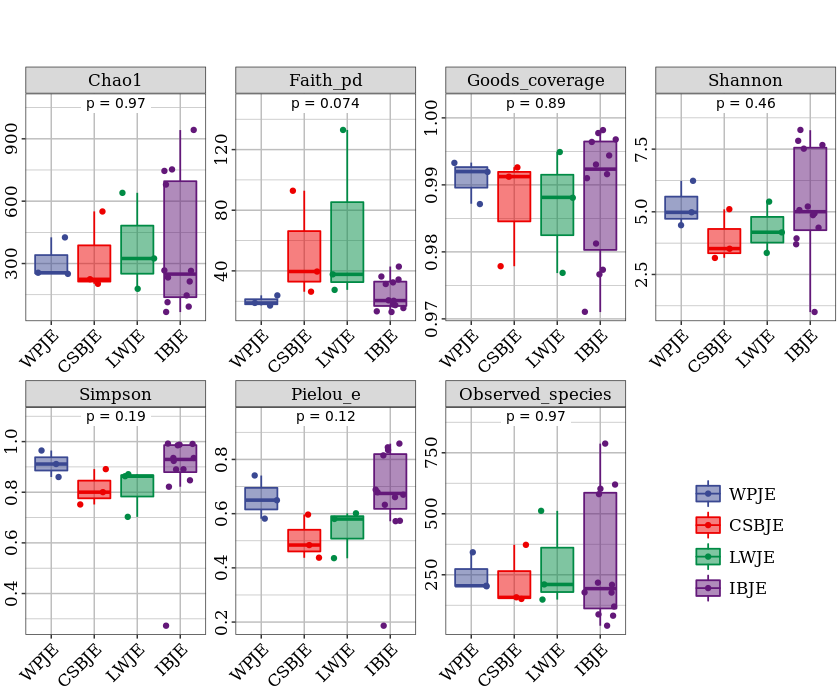

Supplement: Supplementary file 3 — Supplementary file3 (PNG 71 KB) Fig. S3 The alpha-diversity comparisons for the jejunum (JE) in different pig populations. [file 10123_2023_449_MOESM3_ESM.png]

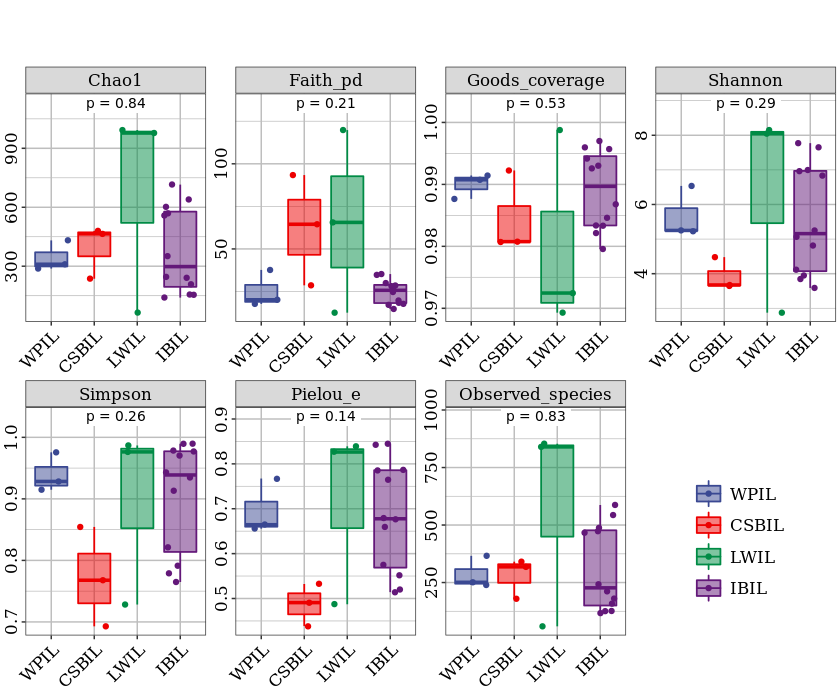

Supplement: Supplementary file 4 — Supplementary file4 (PNG 70 KB) Fig. S4 The alpha-diversity comparisons for the ileum (IL) in different pig populations. [file 10123_2023_449_MOESM4_ESM.png]

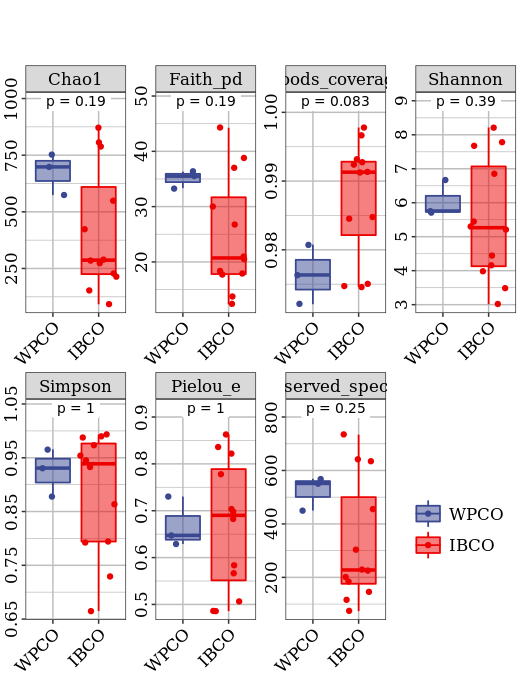

Supplement: Supplementary file 5 — Supplementary file5 (PNG 57 KB) Fig. S5 The alpha-diversity comparisons for the colon (CO) in different pig populations. [file 10123_2023_449_MOESM5_ESM.png]

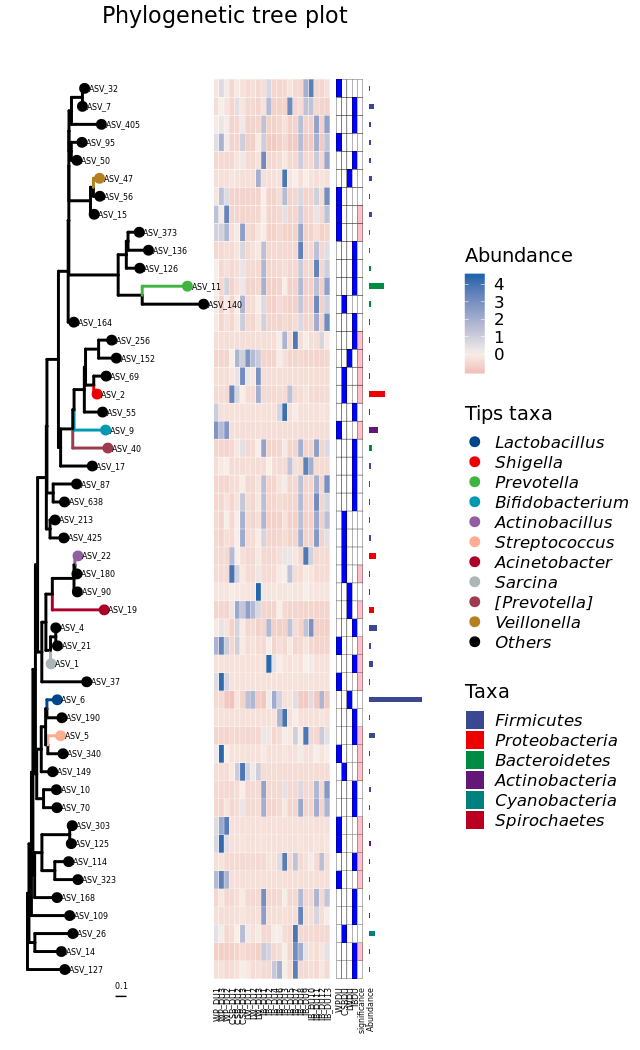

Supplement: Supplementary file 7 — Supplementary file7 (PNG 115 KB) Fig. S7 Phylogenetic tree with ASV abundance distribution in duodenum (DU) of pigs. Species abundance distribution was aligned to the tree and visualized as boxplots. The Phylum information was used to color symbolic points on the tree and also species abundance distributions. [file 10123_2023_449_MOESM7_ESM.png]

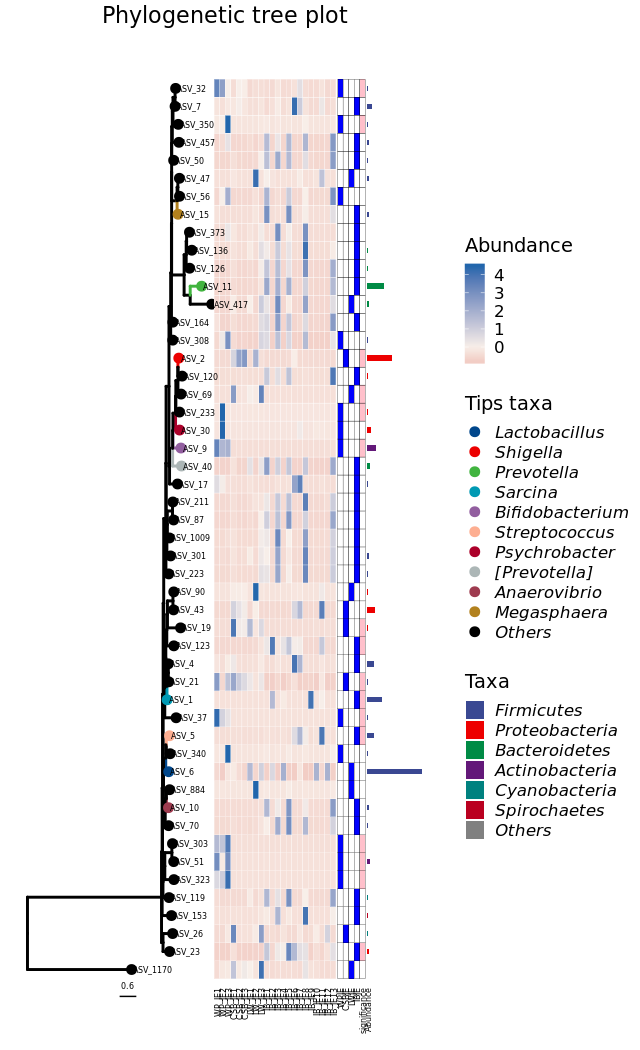

Supplement: Supplementary file 8 — Supplementary file8 (PNG 110 KB) Fig. S8 Phylogenetic tree with ASV abundance distribution in jejunum (JE) of pigs. Species abundance distribution was aligned to the tree and visualized as boxplots. The Phylum information was used to color symbolic points on the tree and also species abundance distributions. [file 10123_2023_449_MOESM8_ESM.png]

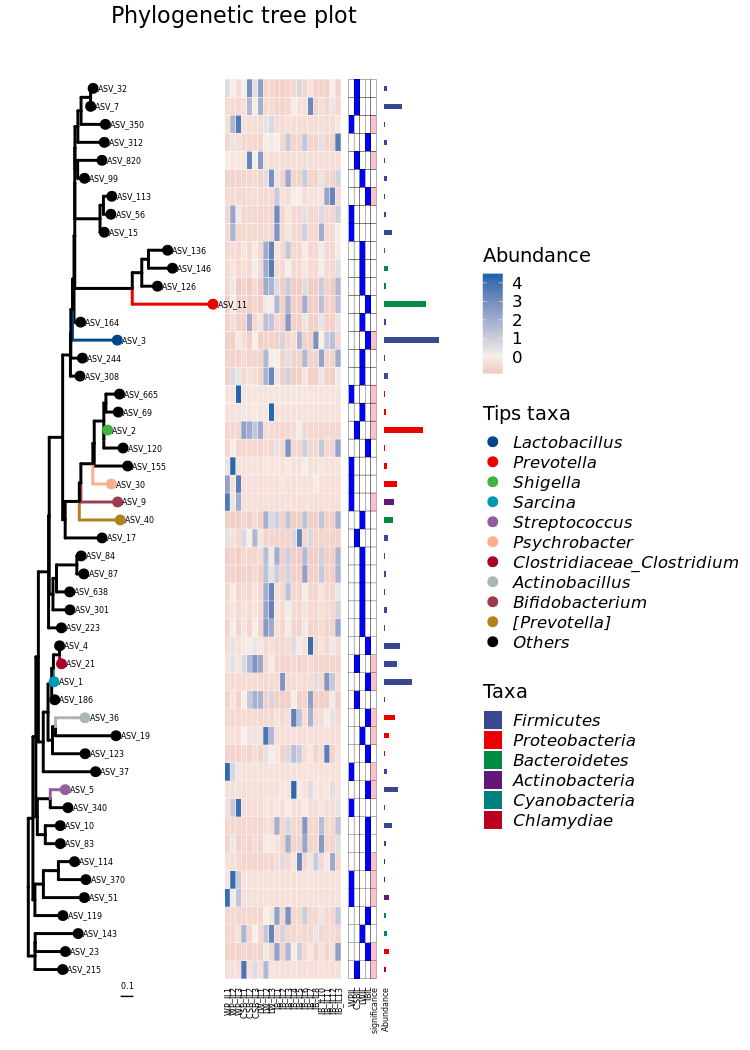

Supplement: Supplementary file 9 — Supplementary file9 (PNG 118 KB) Fig. S9 Phylogenetic tree with ASV abundance distribution in ileum (IL) of pigs. Species abundance distribution was aligned to the tree and visualized as boxplots. The Phylum information was used to color symbolic points on the tree and also species abundance distributions. [file 10123_2023_449_MOESM9_ESM.png]

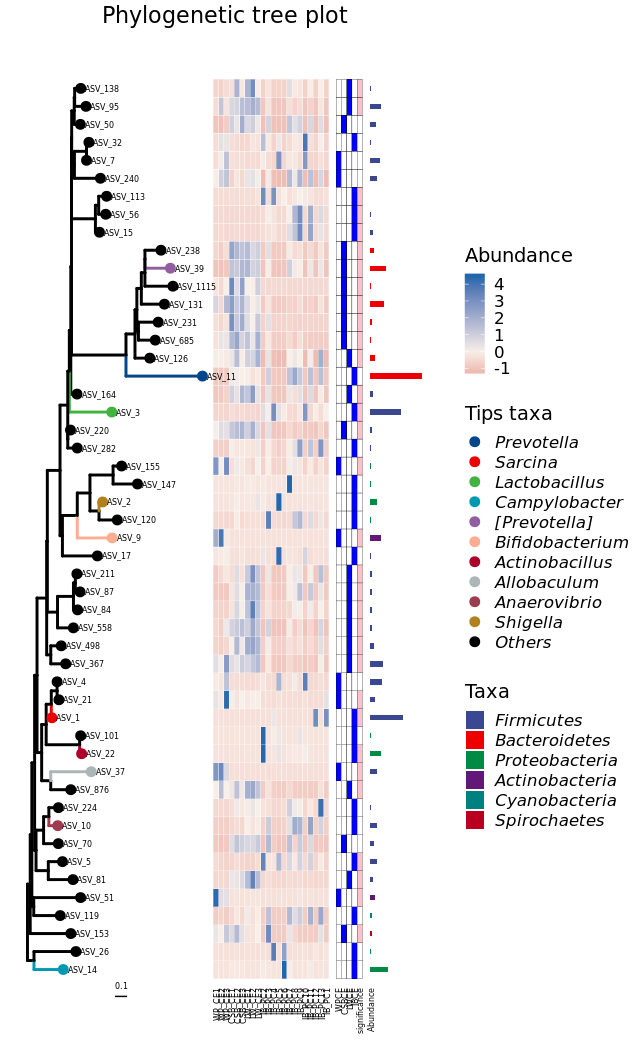

Supplement: Supplementary file 10 — Supplementary file10 (PNG 116 KB) Fig. S10 Phylogenetic tree with ASV abundance distribution in cecum (CE) of pigs. Species abundance distribution was aligned to the tree and visualized as boxplots. The Phylum information was used to color symbolic points on the tree and also species abundance distributions. [file 10123_2023_449_MOESM10_ESM.png]

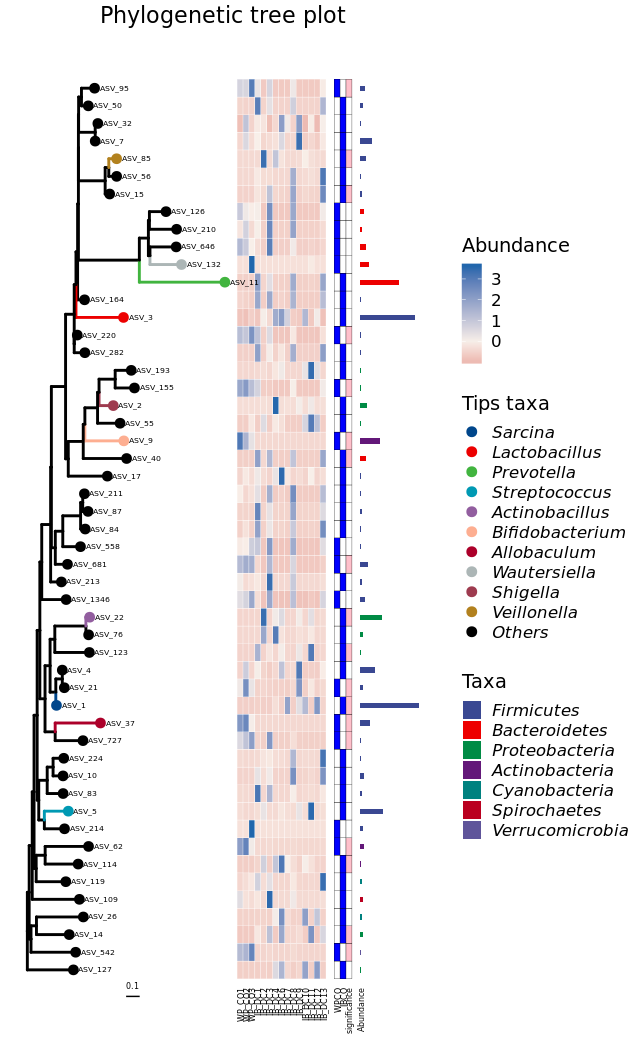

Supplement: Supplementary file 11 — Supplementary file11 (PNG 108 KB) Fig. S11 Phylogenetic tree with ASV abundance distribution in colon (CO) of pigs. Species abundance distribution was aligned to the tree and visualized as boxplots. The Phylum information was used to color symbolic points on the tree and also species abundance distributions. [file 10123_2023_449_MOESM11_ESM.png]
